# Temporal trends of knee osteoarthritis prevalence over a 7-year period in Chinese adults: findings from the CHARLS study 2011–2018

**DOI:** 10.3389/fpubh.2025.1593859

**Published:** 2025-06-11

**Authors:** Ruikang Wang, Junyu Xiang, Muchen Ren, Jianhao Lin

**Affiliations:** ^1^Arthritis Clinical and Research Centre, Peking University People's Hospital, Beijing, China; ^2^Department of Rehabilitation Medicine, Peking University People's Hospital, Beijing, China

**Keywords:** knee osteoarthritis, prevalence, trend, CHARLS, Chinese older adults

## Abstract

**Introduction:**

Knee osteoarthritis (KOA) causes a heavy and increasing burden of disease worldwide. China is facing a significant burden of KOA. However, few studies have investigated the trends of KOA prevalence over time in China using nationwide field-collected data. The study aims to assess the temporal trends of symptomatic KOA prevalence from 2011 to 2018 among Chinese adults aged 45 and older using data from the China Health and Retirement Longitudinal Study (CHARLS).

**Method:**

We conducted a cross-sectional analysis using data from the 2018 wave of CHARLS to assess the prevalence of self-reported symptomatic KOA among participants. Stratified analysis was performed to identify differences across demographic subgroups. Utilizing longitudinal data from the 2011, 2015, and 2018 waves of CHARLS, we further examined the temporal trends in the standardized prevalence of self-reported symptomatic KOA within the overall population and across various demographic subgroups.

**Result:**

Among 19,015 participants form the 2018 wave of CHARLS included in the study, 3,707 (19.5%, 95%CI: 18.87–20.13%) self-reported symptomatic KOA. The prevalence generally increased with age, starting from 10.72% (95% CI: 8.51–12.93%) in the 45–49 age group and peaking at 25.55% (95% CI: 22.86%−28.46%) in the 75–79 age group, before slightly declining to 21.62% (95% CI: 19.00–24.49%) in the oldest age group (>80). Females (24.82%, 95%CI: 23.85–25.82%) exhibited a higher prevalence compared to males (13.64%, 95%CI: 12.89–14.42%). From 2011 to 2018, the standardized prevalence in the overall population increased from 9.86% (95% CI: 9.35–10.38%) to 19.5% (95% CI: 18.87–20.13%), with a rise from 12.48% (95% CI: 11.70–13.31%) to 24.82% (95% CI: 23.86–25.82%) among females and from 6.96% (95% CI: 6.37–7.61%) to 13.64% (95% CI: 12.89–14.42%) among males. Such increase was observed across all demographic subgroups.

**Conclusion:**

The study reveals a double increase in symptomatic KOA prevalence among Chinese adults aged 45 and older from 2011 to 2018. These findings provide new insights into the growing burden of KOA, despite previous research underestimating this trend. Future research on public health policies necessitates more rigorous and comprehensive studies for valuable insights.

## Introduction

Knee osteoarthritis (KOA) is the most common type of osteoarthritis (OA) and the primary cause of knee pain among middle-aged and older adults (aged 45 years and over) (1–4). Lacking effective curative treatments (5), KOA has caused a significant burden of disease worldwide, which has been increasing in recent decades (6, 7). According to a recent study conducted by the Global Burden of Disease (GBD) 2021 Osteoarthritis Collaborators, the global age-standardized prevalence of KOA in 2020 was 4307.4 cases per 100,000 (6). The study also predicted that by 2050, the number of KOA patients worldwide will reach 642 million, representing a 74.9% increase compared to 2020 (6).

China faces a significant burden of KOA (8–10). According to a recent study based on the data from GBD 2019, the incidence and disability-adjusted life years (DALY) rates for OA in China are higher than the average levels in Asia, Africa, and Oceania (8). Given the rapid aging of the population, it is crucial to understand the trends of KOA prevalence in China. Although our previous studies investigated this topic using GBD data (7, 10), the lack of primary research data has limited the validity of their conclusions (11, 12). According to the results of a systematic review, there is a significant lack of available epidemiological data on the prevalence of KOA in China (12). In this context, there is an urgent need for research based on nationally representative field-collected data to fill this gap.

The China Health and Retirement Longitudinal Study (CHARLS) is a comprehensive longitudinal survey of the middle-aged and older adults in China (13). Launched in 2011, CHARLS collects detailed data on the health, socio-economic status, and quality of life of individuals aged 45 and older, with a nationally representative sample across diverse regions and demographic groups (13). Our previous study reported the prevalence of symptomatic KOA among adults aged 45 and older in China based on baseline data from CHARLS (14). Given this background, the current study aims to assess the temporal trends of symptomatic KOA prevalence from 2011 to 2018 among Chinese adults aged 45 and older using data from CHARLS.

## Methods

### Study data source

The CHARLS project aims to provide high-quality micro-data to support research on aging-related issues, including health, retirement, and socioeconomic status (13). The baseline survey was conducted in 2011, covering ~17,500 individuals in 10,000 households across 28 provinces in China (13). Samples were chosen through multistage probability sampling, incorporating a probability-proportional-to-size (PPS) sampling technique at the primary sampling unit level (13, 15, 16). Follow-up surveys were conducted every 2–3 years since baseline, with subsequent waves in 2013, 2015, 2018, and 2020. To maintain representativeness and account for attrition, CHARLS incorporated new sample members in subsequent waves (16). However, the 2020 wave did not include the addition of new, younger participants, which may affect the representativeness of the CHARLS sample for younger age groups (ages 45–46). The CHARLS datasets and its introductory documentation can be downloaded at the CHARLS home page at http://charls.pku.edu.cn/en. Ethical approval for all the CHARLS waves was granted from the Institutional Review Board at Peking University (IRB00001052-11015), and all participants were required to sign informed consent.

### Study population

This study included data from the 2011, 2015, and 2018 waves of the CHARLS project. For the waves included, individuals with incomplete or inconsistent data regarding age, sex, or symptomatic KOA status were excluded from the analysis.

### Assessment of symptomatic KOA

We followed the methodology of our prior study (14) to define symptomatic KOA. Using data from CHARLS, participants were first identified as having knee pain based on their response to the question, “Are you often troubled with any body pains?” If participants answered “yes,” they were further asked to specify the location of the pain. Participants reporting knee pain were then cross-referenced with their response to the question, “Have you been diagnosed with Arthritis or rheumatism by a doctor?” Participants who self-reported knee pain and reported a doctor's diagnosis of arthritis or rheumatism were classified as having symptomatic KOA.

The exclusion of the 2013 wave was due to inconsistencies in the pain assessment. In this wave, pain was evaluated solely on the day prior to the interview, with the question posed as: “Yesterday, did you feel any pain?” This differed from the “frequent pain” definition used in other waves, which asked: “Are you often troubled with any body pains?” This inconsistency compromised data comparability across waves. The 2020 wave was excluded primarily for two reasons: first, the lack of new, younger participants may have introduced bias in the representation of younger age groups; and second, the COVID-19 pandemic may have led to an underestimation of the true prevalence of symptomatic KOA. On one hand, pandemic restrictions limited participants' daily activities (17, 18), potentially masking knee pain symptoms. On the other hand, government lockdown measures increased difficulties in accessing healthcare services, while participants' concerns about infection risk reduced their willingness to seek treatment for knee-related symptoms. These factors may affect the comparability of prevalence estimates with those from other waves.

### Demographic and health characteristics

According to prior knowledge and analytical needs, the following demographic characteristics were included in this study: age (categorized in 5-year intervals from 45–79 and 80+), Sex (Male, Female), Level of Education (No formal education, Elementary school or lower, Junior high school or high school, Vocational school or higher), and Residence Type (Rural, Urban-Rural Fringe, Urban). In addition to these factors, the study included the presence of health conditions such as hypertension, diabetes, Activities of Daily Living (ADL) disabilities, and physical inactivity.

The inclusion of these characteristics aims to investigate two primary objectives. First, it seeks to examine the temporal trends of symptomatic KOA across different subgroups of the population. Second, to investigate whether the temporal trends of symptomatic KOA are associated with shifts in the distribution of these characteristics across the population.

### Assessment of ADL disability

To evaluate ADL disabilities, the study assessed respondents' abilities to perform basic activities of daily living, including dressing, bathing, eating, indoor mobility, toileting, and bowel control, using data collected through the original questionnaires of CHARLS. ADL disabilities were defined as experiencing any difficulties in one or more of these areas.

### Assessment of physical inactivity

For the assessment of physical inactivity, CHARLS employed a questionnaire similar to the International Physical Activity Questionnaire (IPAQ). Respondents reported the frequency and duration of physical activities categorized into Light Physical Activity (LPA), Moderate Physical Activity (MPA), and Vigorous Physical Activity (VPA) on a weekly basis. Frequency was defined as the number of days per week on which respondents participated in physical activity lasting at least 10 min, while duration was categorized into four levels: 10–29 min, 30–119 min, 120–239 min, and 240 min or more. Due to a lack of specific values, median values were used for each duration category based on previous study (19), with the “240 min or more” group considered as 240 min. The overall level of physical activity was calculated by multiplying frequency, duration, and the assigned MET (Metabolic Equivalent of Task) values for each intensity, which were defined as 3.3 MET for LPA, 4 MET for MPA, and 8 MET for VPA according to prior research (20). Following WHO and related guidelines (21, 22), a physical activity level of less than 600 MET-min/week was classified as physical inactivity.

In the 2011 and 2015 waves, the survey on physical activity was conducted only among half of the randomly selected respondents. In contrast, all respondents in the 2018 wave participated in this survey.

### Statistical analysis

Descriptive statistics were used to illustrate the composition of demographic and health characteristics among participants in the three included waves. Categorical variables were summarized using frequency counts and proportions (*n*, %), while continuous variables were presented as means and standard deviations (SD). Additionally, chi-square tests were conducted to assess the distributional differences of each characteristic among the different waves of the population.

In the prevalence analysis, we first utilized the cross-sectional data from the 2018 wave to investigate the prevalence of symptomatic KOA across different age groups. Specifically, we calculated the prevalence of symptomatic KOA in each age group, stratified by sex, and conducted chi-square tests to evaluate the differences. Subsequently, we calculated the standardized prevalence of symptomatic KOA for the overall population and for subgroups defined by demographic and health characteristics across all waves to describe the trends in prevalence over time. Considering that increased awareness of KOA and improved access to medical services may lead to an increase in self-reported prevalence and potentially obscure the observation of true temporal trends in KOA prevalence (23), we also investigated trends in the prevalence of knee pain over time using the same analytical procedures to validate the reliability of our findings.

The 2018 wave population was selected as the reference population for prevalence standardization to more intuitively present the true prevalence rates at the most recent time point. The standardization was conducted by age and sex. For age subgroups, prevalence rates were standardized by sex composition only. Similarly, for sex-specific subgroups, prevalence rates were standardized by age composition only. For all prevalence estimates, 95% confidence intervals (CIs) were reported. Informed by previous studies by Woolson and Bean (24), we applied the Mantel-Haenszel chi-square test to evaluate the significance of trends in standardized prevalence. Additionally, the assessment of 95%CI was also employed to evaluate the significance of differences in the standardized prevalence rates, where non-overlapping 95%CIs suggest statistically significant differences between the prevalence rates.

nivariate (chi-square tests) and multivariate (logistic regression) analysis were conducted based on the data from 2018 wave to investigate the correlation between demographic/health characteristics and the status of KOA. To further investigate the contribution of various characteristics to the observed temporal change in KOA prevalence, we used the identified associated factors as explanatory variables to conduct a Blinder-Oaxaca decomposition (25, 26) analysis on the differences in symptomatic KOA prevalence between the 2011 and 2018 waves.

Blinder-Oaxaca decomposition is a method used to explain the difference in the level of a dependent variable (KOA status) between two groups (2011 wave and 2018 wave) by decomposing the gap into two parts (27). One part is attributed to differences in the levels of explanatory variables (demographic and health characteristics associated to symptomatic KOA status) between the groups, while the other part accounts for differences in the effects of these explanatory variables (the magnitude of regression coefficients) as well as other unknown associated factors (27).

In this study, logistic regression models were constructed separately for 2011 and 2018 group. Then we conducted a two-fold Blinder-Oaxaca decomposition to analyze the contributions of included factors to the differences in KOA prevalence between groups. The contribution of each factor to the explained difference was estimated using the equally weighted average of the coefficients from the two models, a method that was initially introduced by Reimers (28). The ratio of each included characteristic's contribution to the total difference was calculated and reported, respectively.

Data missingness was reported in the descriptive statistics. For the dataset used in the Blinder-Oaxaca decomposition analysis, we utilized Multiple Imputation by Chained Equations (MICE) (29) to handle missing values. For all other analysis, only eligible individuals with complete data were included.

A two-sided alpha level of 0.05 was used for all statistical significance tests, with *P* < 0.05 considered statistically significant. All analysis were performed using R version 4.1.0. The standardization of prevalence was performed using the package “epitools.” The chi-square test and Mantel-Haenszel chi-square test were performed using package “stats.” The Blinder-Oaxaca decomposition was performed using package “oaxaca”.

## Results

### Demographic and health characteristics

**Figure 1:**
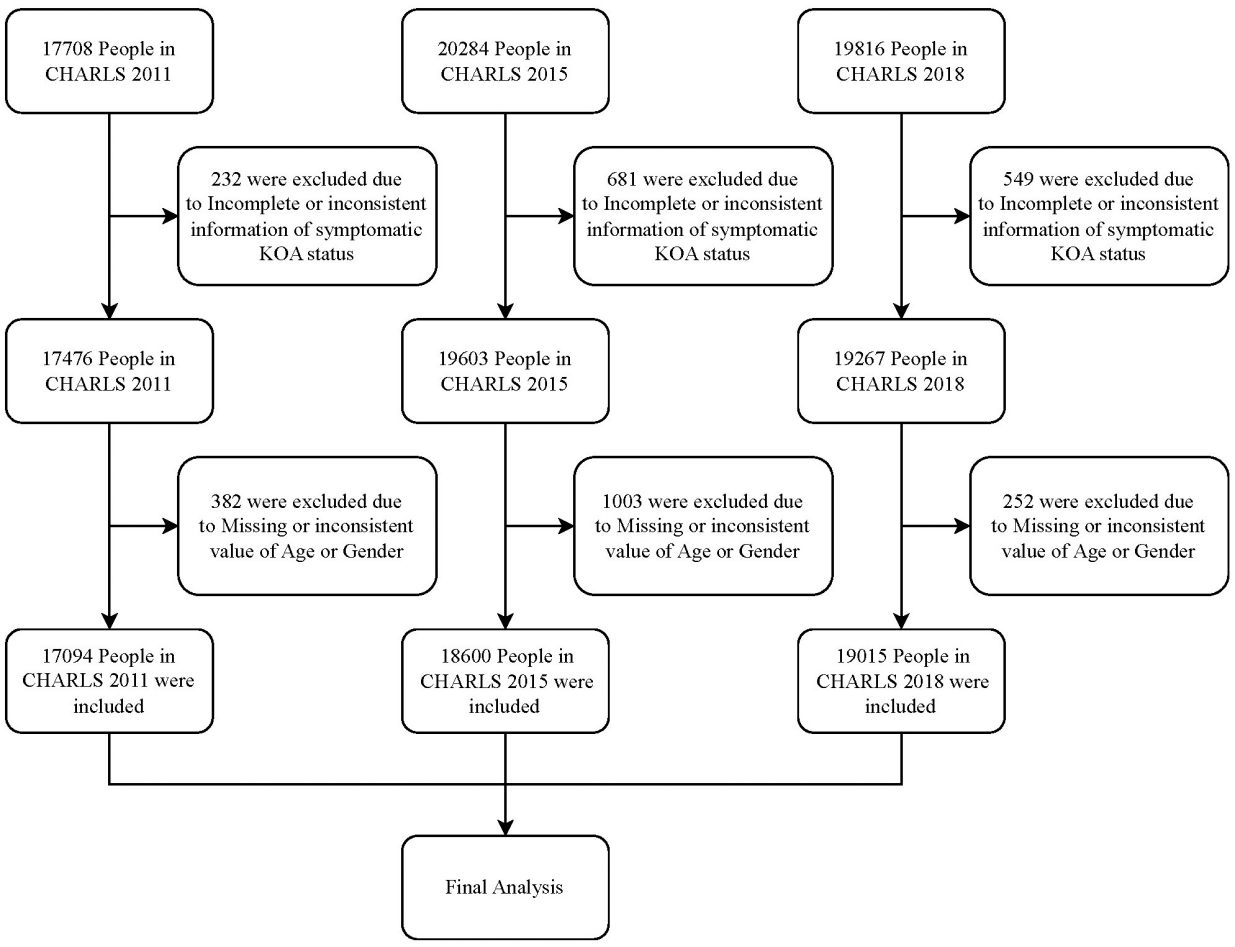
Flow chart for enrollment of study participants.

The final sample comprised 17,094, 18,600, and 19,015 participants from the 2011, 2015, and 2018 waves of the CHARLS project, respectively. The detailed sample selection process is illustrated in Figure 1.

The mean age of the participants was 59.02 years (SD = 10.13) in 2011, 59.2 years (SD = 10.4) in 2015, and 61.75 years (SD = 10.42) in 2018. The sex distribution was relatively balanced, with ~48–49% male and 51–52% female participants. Approximately 70% of participants resided in rural areas, and <5% had completed vocational school or higher education.

Chi-square tests indicated that between the 2011 and 2018 waves, there was an increase in the proportion of older individuals (*P* < 0.001), those with higher education levels (*P* < 0.001), and residents of urban-rural fringes (from 1.42% in 2011 to 8.16% in 2018, *P* < 0.001). Additionally, the prevalence of hypertension (from 24.75% in 2011 to 37.95% in 2018, *P* < 0.001), diabetes (from 5.81% in 2011 to 12.31% in 2018, *P* < 0.001), and ADL disabilities (from 16.73% in 2011 to 18.74% in 2018, *P* < 0.001) showed a marked increase, whereas the prevalence of physical inactivity significantly decreased (from 18.72% in 2011 to 16.08% in 2018, *P* < 0.001). The sex composition of the participants showed no significant changes across the waves (*P* = 0.102). Detailed demographic/health characteristics of the study population are summarized in Table 1.

**Table 1:** Demographic characteristics of the study population.

**Characteristics**	**2011 (*****n*** = **17,094)**		**2015 (*****n*** = **18,600)**		**2018 (*****n*** = **19,015)**		P^*^
	**Sample (** ***n*** **)**	**Proportion (%)**	**Sample (** ***n*** **)**	**Proportion (%)**	**Sample (** ***n*** **)**	**Proportion (%)**	
Age group							<0.001
45–49	3,454	20.21	2,951	15.87	1,931	10.16	
50–54	2,549	14.91	3,512	18.88	3,422	18	
55–59	3,512	20.55	2,770	14.89	2,966	15.6	
60–64	2,875	16.82	3,468	18.65	3,287	17.29	
65–69	1,828	10.69	2,544	13.68	3,063	16.11	
70–74	1,363	7.97	1,689	9.08	1,924	10.12	
75–79	867	5.07	1,009	5.42	1,284	6.75	
>80	646	3.78	657	3.53	1,138	5.98	
Sex							0.102
Male	8,327	48.71	9,000	48.39	9,056	47.63	
Female	8,767	51.29	9,600	51.61	9,959	52.37	
Level of education							<0.001
No formal education	4,668	27.34	4,183	25.63	4,299	22.61	
Primary education or below	6,696	39.22	6,673	40.89	8,118	42.69	
Secondary education	4,877	28.57	4,714	28.89	5,743	30.2	
Vocational education or higher	832	4.87	749	4.59	855	4.5	
Missing value	21	Not included	2,281	Not included		
Residence type							<0.001
Rural	12,676	74.31	12,906	69.96	13,534	71.53	
Urban-Rural fringe	243	1.42	1,707	9.25	1,543	8.16	
Urban	4,139	24.26	3,834	20.78	3,843	20.31	
Missing value	36	Not included	153	Not included	95	Not included	
Hypertension							<0.001
No	12,796	75.25	12,645	68.86	11,651	62.05	
Yes	4,208	24.75	5,718	31.14	7,127	37.95	
Missing value	90	Not included	237	Not included	237	Not included	
Diabetes							<0.001
No	15,957	94.19	16,887	91.25	16,549	87.69	
Yes	985	5.81	1,619	8.75	2,323	12.31	
Missing value	152	Not included	94	Not included	143	Not included	
ADL disability							<0.001
No	14,056	83.27	14,573	80.71	15,250	81.26	
Yes	2,824	16.73	3,483	19.29	3,518	18.74	
Missing value	214	Not included	544	Not included	247	Not included	
Physical inactivity							<0.001
No	5,385	81.28	7,490	82.93	15,954	83.92	
Yes	1,240	18.72	1,542	17.07	3,056	16.08	
Missing value	10,469	Not included	9,568	Not included	5	Not included	

^*^P value was calculated using chi-square test. A *P* value < 0.05 is considered statistically significant, indicating meaningful differences between subgroups of the corresponding demographic characteristics. CI, Confidence Interval.

### Age-related differences in the prevalence of symptomatic KOA

**Figure 2:**
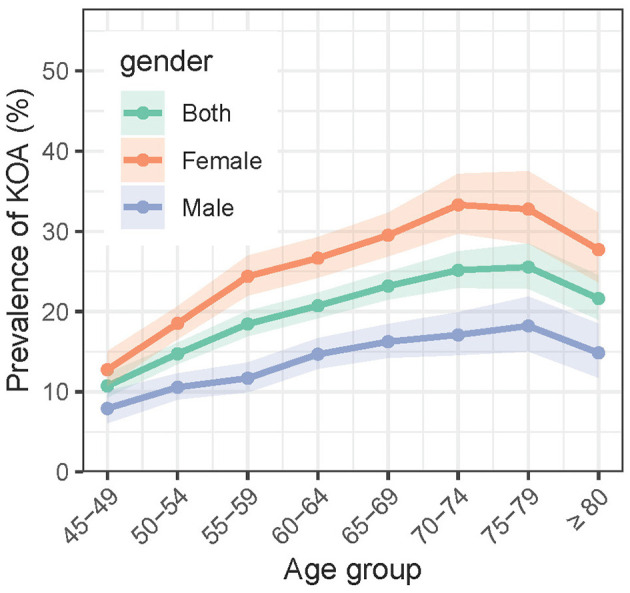
Age specific prevalence of symptomatic KOA in 2018.

In the 2018 wave, among all the 19,015 participants, 3,707 (19.5%, 95%CI: 18.87–20.13%) self-reported symptomatic KOA. Figure 2 shows the age trends of symptomatic KOA prevalence stratified by sex. The prevalence of symptomatic KOA generally increases with age. For the overall population, the prevalence started from 10.72% (207/1931, 95% CI: 8.51–12.93%) in the 45–49 age group and peaked at 25.55% (328/1284, 95% CI: 22.86%−28.46%) in the 75–79 age group (*P* < 0.001), before slightly declining to 21.62% (246/1138, 95% CI: 19.00–24.49%) in the oldest age group (>80) (*P* = 0.026). The trend for males was similar to that of the overall population, with the prevalence starting at 7.91% (64/809, 95% CI: 6.09–10.10%) in the 45–49 age group, peaking at 18.21% (116/637, 95% CI: 15.05–21.84%) in the 75–79 age group (*P* < 0.001), and declining to 14.84% (80/539, 95% CI: 11.77–18.47%) in the oldest age group (>80) (*P* = 0.142). However, notably, the prevalence of symptomatic KOA among females, which started at 12.75% (143/1122, 95% CI: 10.74–15.01%) in the 45–49 age group, peaked earlier in the 70–74 age group, reaching 33.30% (319/958, 95% CI: 29.74–37.16%) (*P* < 0.001), and then declined to 27.71% (166/599, 95% CI: 23.66–32.26%) in the oldest age group (>80) (*P* = 0.053). Females exhibited a higher prevalence of symptomatic KOA compared to males across all age groups (*P* < 0.001).

### Temporal trends of prevalence

**Figure 3:**
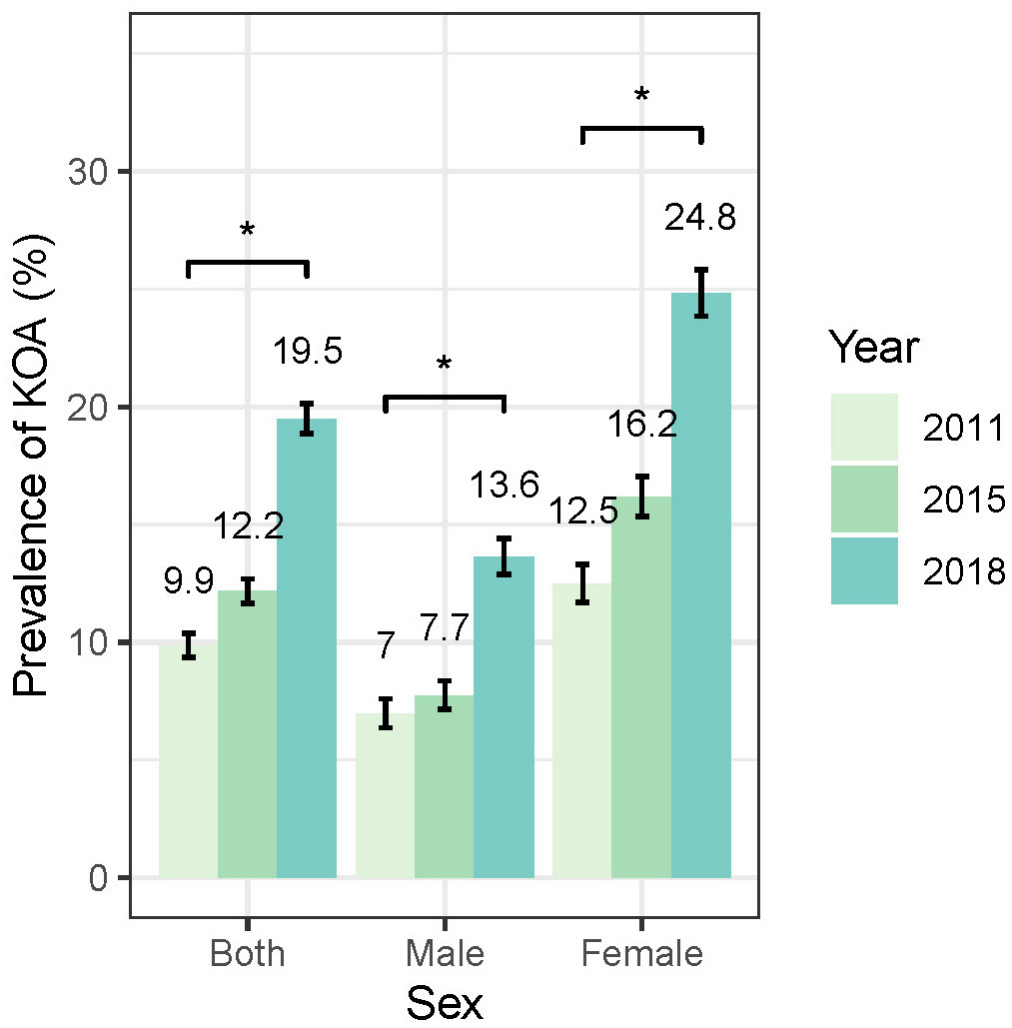
Temporal trends in standardized KOA prevalence stratified by sex.

**Figure 4:**
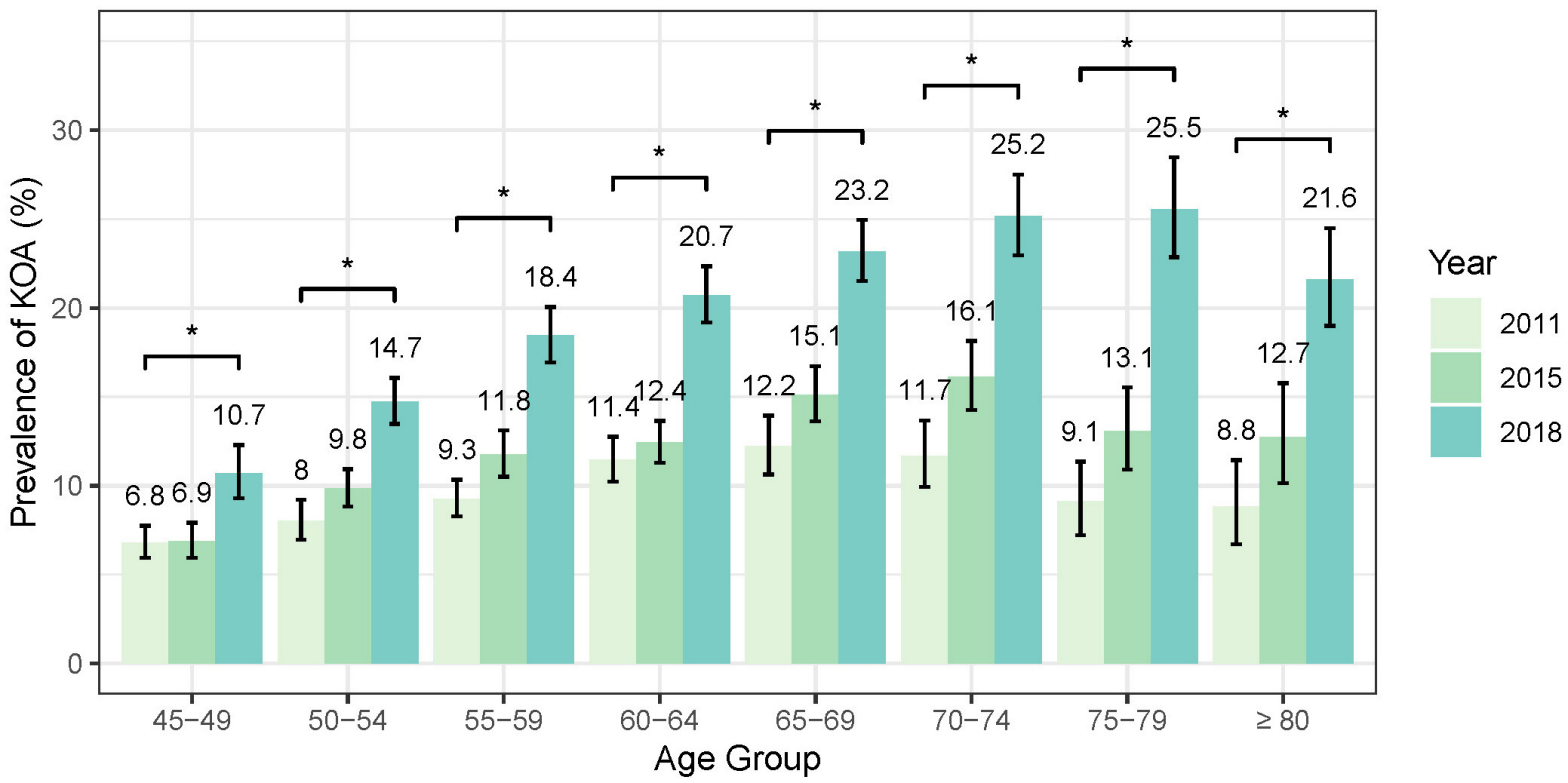
Temporal trends in standardized KOA prevalence stratified by age.

As shown in Figure 3, the standardized prevalence of symptomatic KOA in the overall population showed a marked increase from 9.86% (95% CI: 9.35–10.38%) in 2011 to 12.16% (95% CI: 11.65–12.7%) in 2015 and reaching 19.5% (95% CI: 18.87–20.13%) in 2018. Similar increasing trends were observed in both female and male populations. The standardized prevalence of symptomatic KOA among female population started at 12.48% (95% CI: 11.70–13.31%) in 2011 and increased to 24.82% (95% CI: 23.86–25.82%) in 2018, whereas in male population the prevalence started at 6.96% (95% CI: 6.37–7.61%) in 2011 and increased to 13.64% (95% CI: 12.89–14.42%) in 2018. Notably, the prevalence of symptomatic KOA was higher in female than in male population at each follow-up (Figure 3, Supplementary Table 1). Stratified analysis by age indicates that the prevalence of symptomatic KOA has significantly increased within the study period across all age subgroups (Figure 4, Supplementary Table 1). Similar trends in prevalence were also observed for knee pain (Supplementary Table 2). More details are available in Supplementary Tables 1, 2.

### Temporal trends of prevalence in subgroups

**Figure 5:**
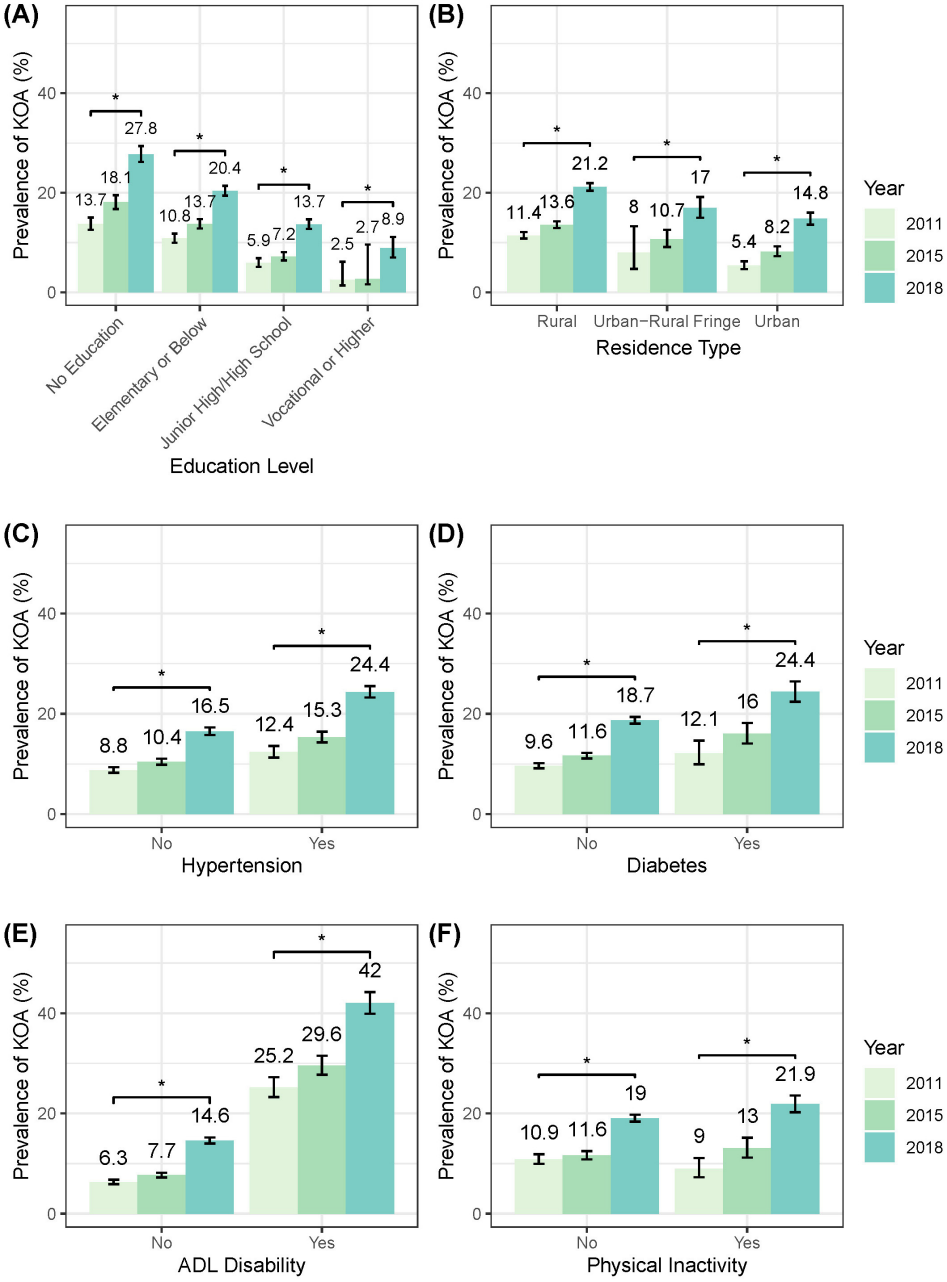
Temporal trends in standardized KOA prevalence stratified by sociodemographic/health characteristics. **(A)** By education level. **(B)** By residence type. **(C)** By hypertension status. **(D)** By diabetes status. **(E)** By ADL status. **(F)** By level of physical activity. ADL, activity of daily living.

The stratified analysis revealed a consistent pattern of increasing prevalence of symptomatic KOA across all demographic/health subgroups from 2011 to 2018 (Figure 5, Supplementary Table 1). The parallel analysis of knee pain showed that the prevalence trends of knee pain were similar to those observed for symptomatic KOA across all subgroups (Supplementary Table 2).

### Associated factors of symptomatic KOA and their contribution to temporal changes in prevalence

The chi-square test conducted on the 2018 wave data revealed that all the demographic/health characteristics were significantly associated with symptomatic KOA status. Specifically, the prevalence of symptomatic KOA was significantly higher among older adults (*P* < 0.001), females (*P* < 0.001), individuals with lower education levels (*P* < 0.001), and those residing in rural areas (*P* < 0.001). Additionally, the prevalence was notably elevated in individuals with hypertension (*P* < 0.001), diabetes (*P* < 0.001), ADL disabilities (*P* < 0.001), and physical inactivity (*P* < 0.001). In contrast, in the multivariate logistic regression model, the association between age and symptomatic KOA status was no longer significant (*P* = 0.865). More importantly, the presence of physical inactivity demonstrated a significantly negative association with the occurrence of symptomatic KOA (*P* < 0.001). Further examination revealed that the reversal of the association between physical inactivity and symptomatic KOA status occurred when ADL status was included in the regression model. The chi-square test demonstrated a significant positive association between the presence of physical inactivity and the existence of ADL disabilities. Additionally, a significant interaction between ADL disabilities and physical inactivity was observed in the regression model. Subgroup analysis indicated that the negative association effect of physical inactivity with symptomatic KOA was more pronounced in the population with ADL disabilities.

The significant associated factors identified from the multivariate regression analysis were incorporated into the Blinder-Oaxaca decomposition analysis. The results indicated that changes in the prevalence of hypertension between the 2011 and 2018 waves accounted for 5.66% of the differences in KOA prevalence, which was the highest among all factors included. Following hypertension, the next factors contributing to the prevalence differences in order were ADL status (4.76%), diabetes status (1.34%), physical activity (0.73%), type of residence (0.12%), and education level (−2.35%). A negative ratio indicates that the contribution of the factor is in the opposite direction of the overall difference.

## Discussion

This study aimed to investigate the temporal trends of symptomatic KOA prevalence from 2011 to 2018 among Chinese middle-aged and older adults (aged 45 years and older) using data from CHARLS. Our findings revealed a significant upward trend in the standardized prevalence of symptomatic KOA, nearly doubling from 9.86% in 2011 to 19.5% in 2018. Stratified analysis by age, sex, and other demographic or health characteristics indicated that the prevalence of symptomatic KOA displayed a similar increasing temporal trend across all subgroups of the population. This alarming trend underscores the escalating burden of KOA in China and highlights the urgent need for effective prevention and intervention strategies, especially considering the country's large population (9).

Previous studies conducted by us (7, 10) and other researchers (8) have reported trends in the prevalence and disease burden of OA/KOA in China over the past few decades, utilizing data from the GBD study. According to a prior study conducted by us (10), the age-standardized prevalence of OA in China increased from 2.9% in 1990 to 3.1% in 2017. Another study (7) reported that the age-standardized prevalence of KOA in China increased from 4.8% in 1990 to 5.13% in 2019. The observed rising trend in prevalence from these studies is consistent with our findings; however, the specific rate of increase is markedly lower than the results observed in this study. Although differences in study populations, case definitions, data sources, and standardization methods may partially account for this discrepancy, it is important to acknowledge that the inherent limitations within GBD data itself may also affect the accuracy of previous research findings and contribute to this inconsistency (11, 30).

Despite being considered the largest and most comprehensive global epidemiological study to date, the Global Burden of Disease (GBD) still has inherent limitations in its investigation of OA (11). First, the data provided by GBD are derived from aggregated estimates of original studies (6), and the scarcity of primary data restricts the validity of the findings (11). Sun et al. (12) conducted a systematic review of epidemiological research evidence on OA in China and concluded that there is a significant scarcity of OA epidemiological data, as most existing studies are regionally focused and of small scale, and there is considerable variability in the prevalence rates reported across different investigations. Second, another issue is the inconsistency in the definition of OA across the original studies; for instance, some studies relied on radiographic diagnoses of OA, while others investigated self-reported cases of the condition (6, 11). Furthermore, the GBD study employed a modeling-based approach to conduct a meta-analysis of the included original studies and to estimate disease burden and prevalence (31, 32). The estimation process was carried out in a hierarchical order, including global, super-region, region, country, and subnational location. The fitting results from each higher level provided prior information for subsequent lower-level fitting processes (31, 32). This methodology implies that, in estimating the prevalence in the Chinese population, data from East Asia, Southeast Asia, and even other global contexts are referenced. On one hand, this approach helps to address gaps caused by sparse original data; on the other hand, it may introduce bias due to differing national contexts. Over the past few decades, China has experienced significant changes in its socioeconomic structure and demographic composition, which may further complicate the applicability of findings derived from other regions. All these limitations have restricted the ability of the GBD study to accurately reflect the prevalence of KOA in China and may have led to an underestimation of the true rate of increase in KOA prevalence.

In contrast, the data utilized in this study comes from a nationally representative field survey, which employs a consistent and objective data collection process across different time points (16). This enhances the longitudinal comparability of the data, allowing for a more accurate and comprehensive assessment of the trends in KOA prevalence nationwide, while being less influenced by potential confounding factors, such as policy changes. In this context, we suggest that the prevalence trend observed in this study may offer a revision to the findings of previous research, indicating that earlier studies could have underestimated the growth trend of KOA prevalence in China.

Increased awareness of KOA and improved access to medical services may lead to an increase in self-reported disease prevalence (23), thereby obscuring the accurate estimation of true prevalence trends, as more symptomatic individuals seek medical care and receive a diagnosis. To strengthen the persuasiveness of our findings, we investigated the trends in self-reported prevalence of knee pain, which may indirectly reflect the true prevalence trends of KOA while being less influenced by the aforementioned factors. The results demonstrated that the standardized prevalence of knee pain in the overall population increased from 13.6% in 2011 to 29.09% in 2018, which was a growth rate comparable to that of symptomatic KOA prevalence. Furthermore, the trend in the standardized prevalence of knee pain symptoms across various subpopulations mirrored that of symptomatic KOA. This result suggests that while increased awareness and access to medical services may play a role, they are unlikely to be the primary drivers behind the observed increase in KOA prevalence in our study, which strengthens the reliability of our main findings.

Another study (33) supports our opinion indirectly. Chen et al. (33) utilized claims data from the Beijing Medical Claim Data for Employees (BMCDE), which includes health insurance records of 17.7 million adults in Beijing, to identify KOA patients and investigate the trends in prevalence. Their results indicated that the age-standardized prevalence of KOA among adults in Beijing increased from 1.57% in 2008 to 5.94% in 2017 (33). Similar to our findings, the rate of increase in KOA prevalence observed in Chen et al.'s study was much higher than that reported in previous research that utilized GBD data. However, the study by Chen et al. (33) also had limitations. As they stated, the reliance on hospital visit data may lead to an underestimation of the true prevalence, and the use of ICD coding to determine disease status might be influenced by the diagnostic process.

The underestimation of the true prevalence trends may lead to misguided allocation of healthcare resources and ineffective public health interventions. Therefore, research on more rigorous and comprehensive approaches to this topic is needed to provide valuable insights into the formulation of public health policies. Additionally, it is essential to conduct further investigations into the factors influencing trends in KOA prevalence, which will enable us to better understand the dynamics of this condition and develop targeted strategies to effectively address the increasing burden of KOA.

The reliance on self-reported diagnoses may lead to an underestimation of the true prevalence of the condition, particularly among rural populations, individuals with lower education levels, and older adults. These groups often face greater challenges in understanding the disease and accessing medical services. In light of these considerations, we propose that future studies of this nature should incorporate a methodology that allows respondents to self-diagnose based on specific criteria for typical cases, such as the presence of brief morning stiffness, significant joint deformity, limited joint mobility, and pain symptoms. This approach could help to reduce statistical bias and improve the accuracy of prevalence estimates.

This study also provides additional findings. Our results indicate that the prevalence of symptomatic KOA increases progressively with age, peaking in the 70–79 age group before declining in the older cohorts. Although there may be slight variations in the age group corresponding to the peak prevalence, this trend aligns with findings from a few previous domestic (33) and international (6, 34) studies. A potential explanation for the observed decline in the prevalence among older populations is that, when assessing the prevalence of symptomatic KOA in older age groups, the underestimation due to survivorship bias may outweigh the cumulative risk of developing the disease associated with longer survival. Previous studies have widely recognized the positive association between knee osteoarthritis (KOA) and mortality risk (35, 36), as well as other health conditions (37–41). On one hand, KOA is frequently comorbid with various chronic conditions that elevate mortality risk, such as hypertension (37, 38), diabetes (39, 40), and cardiovascular disease (CVD) (41). On the other hand, KOA itself can lead to functional impairment and disability (1). We hypothesize that, over time, the disparities in the prevalence and severity of these health conditions between KOA patients and non-patients may gradually increase due to the accumulation of risk and disease progression. Consequently, the widening gap in mortality risk may become more pronounced, resulting in a stronger effect of KOA on mortality risk in older populations compared to younger individuals. This scenario, coupled with a higher baseline mortality risk, may lead to a more significant survivorship bias among older populations, thereby explaining the observed decline in the prevalence of KOA. This inference may also help explain the phenomenon observed in previous study, where the prevalence of OA in joints other than the knee monotonically increases with age (6). This is likely because OA in other locations generally exerts a less significant impact on health compared to KOA, resulting in a weaker survivorship bias. Notably, the results of the multifactorial analysis suggest that the correlation between age and symptomatic KOA is primarily mediated by other factors included in this study. In other words, the unequal distribution of various characteristics is the main reason for the differing prevalence of symptomatic KOA among different age groups.

Additionally, previous studies have reported a higher prevalence of symptomatic KOA in female than in male population (6, 14, 34, 42), which was also observed in this study. The mechanisms underlying the sex differences in knee osteoarthritis (KOA) are not yet fully understood. However, it is currently believed that the significant decline in estrogen levels after menopause is associated with the rapid increase in KOA prevalence among female population in this age group, as estrogen is considered to play a protective role against cartilage degeneration (43). Additionally, this phenomenon may also be partially attributed to differences in quadriceps strength, lower limb biomechanics, and molecular genetics between the sexes (43). Furthermore, a higher proportion of female KOA patients experience symptoms compared to their male counterparts, which may be related to differences in pain sensitivity between sexes (43). We also noted significant disparities in the prevalence of KOA among other demographic subgroups. Specifically, patients with symptomatic KOA were more densely distributed among populations with lower education levels and those residing in rural areas. These findings corroborate the conclusions of earlier studies (42, 44–47), which may reflect the underlying differences in socioeconomic status, access to healthcare, and lifestyle factors. Previous studies have confirmed a negative correlation between socioeconomic status and KOA (46, 48). Potential reasons for this correlation include differences in obesity prevalence (49), self-efficacy (50) and level of physical labor (51) among various socioeconomic groups. Additionally, disparities in healthcare accessibility between urban and rural areas may reduce medical support for joint injuries and degenerative changes in rural populations, thereby contributing to the observed differences in KOA prevalence.

This study also observed a correlation between health characteristics and symptomatic KOA. To be specific, the prevalence of symptomatic KOA is higher among individuals with hypertension, diabetes, ADL disability, and physical inactivity. However, according to the results of the multifactorial analysis, the significant positive association between the presence of physical inactivity and ADL disability appears to be the primary reason for the higher prevalence of symptomatic KOA in the physical inactivity population. After stratifying by ADL status, physical inactivity showed a negative association with the presence of symptomatic KOA, and this effect was stronger in the population with ADL disabilities. One possible explanation for this phenomenon is that decreased physical activity reduces the load on the knees in the population, which may mask the symptoms of knee pain and lead to a lower prevalence of symptomatic KOA. At the same time, the stronger association observed in individuals with ADL disabilities may be related to their greater sensitivity to knee joint load, as this population may have a higher prevalence of knee joint pathology and lower limb muscle insufficiency.

According to the results of this study, changes in the distribution of characteristics within the population during the study period—mainly the increase in the prevalence of hypertension and ADL disabilities—partially explain the rise in the prevalence of symptomatic KOA. However, this accounts for only a small portion of the overall increase in prevalence, with most of the effects remaining unexplained by the factors included in the study. As an important risk factor for KOA (51), the prevalence of obesity continues to rise among Chinese residents, which may contribute to the observed trends of prevalence in this study. Due to limitations in the original data, this study could not investigate the role of obesity in the increase of symptomatic KOA prevalence. Further research on this topic is warranted. Caution should be exercised in interpreting these results, as the cross-sectional design of this study does not eliminate the possibility of reverse causation. It is possible that the observed increase in the prevalence of hypertension and ADL disabilities within the population may, in fact, be a consequence rather than cause of the rising prevalence of symptomatic KOA.

There are several notable advantages in this study. First, the data collection was conducted through field surveys, with consistent standards and procedures maintained across different time points. This ensures the authenticity of the observed prevalence and their comparability over time, thereby enhancing the reliability of the study's conclusions. Second, the study benefits from a large sample size sourced from multiple regions nationwide, generated through rigorous random sampling, which provides excellent representativeness of the middle-aged and older adult population in China. Lastly, the use of individual-level data enables the analysis of the roles of various demographic and health characteristics in the temporal trends of symptomatic KOA prevalence.

This study had several limitations. First, the definition of symptomatic KOA in this study was based on participants' self-reported knee pain' combined with “having been diagnosed with Arthritis or rheumatism by a doctor.” This may lead to misclassification, potentially resulting in an overestimation of the KOA prevalence within population. However, we believe that this error has a limited impact on the internal comparability of prevalence rates across different periods and populations. Additionally, the prevalence trends derived from self-reported physician diagnoses may be influenced by the level of disease awareness in the population and availability of healthcare resources. Although we included an investigation into the trends of knee pain prevalence, this approach can only provide an indirect assessment and cannot entirely eliminate such confounding factors. Therefore, the findings of this study should be interpreted with caution, especially concerning the absolute values of prevalence rates.

## Conclusion

The study reveals a double increase in symptomatic KOA prevalence among Chinese adults aged 45 and older from 2011 to 2018. These findings provide new insights into the growing burden of KOA, despite previous research underestimating this trend. Future research on public health policies necessitates more rigorous and comprehensive studies for valuable insights.

## Data Availability

The original contributions presented in the study are publicly available. This data can be found here: http://charls.pku.edu.cn/en.
