# Supplementary material for: Temporal trends of knee osteoarthritis prevalence over a 7-year period in Chinese adults: findings from the CHARLS study 2011–2018
Source: Front Public Health. 2025 Jun 11;13:1593859. doi: 10.3389/fpubh.2025.1593859 (PMC12187600; doi:10.3389/fpubh.2025.1593859)
Supplement: Supplementary file 1 [file Data_Sheet_1.pdf]

Supplementary Table 1. Temporal trends of standardized KOA prevalence across demographic/health subgroups

| **Characteristics** | **2011 (*n* = 17094)** | | | | **2015 (*n* = 18600)** | | | | **2018 (*n* = 19015)** | | | |
|---|---|---|---|---|---|---|---|---|---|---|---|---|
| | **Case (*n*)** | **Pop (*n*)** | **Crude prevalence (%) [95% CI]** | **Standardized prevalence (%) [95% CI]** | **Case (*n*)** | **Pop (*n*)** | **Crude prevalence (%) [95% CI]** | **Standardized prevalence (%) [95% CI]** | **Case (*n*)** | **Pop (*n*)** | **Crude prevalence (%) [95% CI]** | **Standardized prevalence (%) [95% CI]** |
| Age group | | | | | | | | | | | | |
| 45-49 | 230 | 3454 | 6.66 (5.83-7.58) | 6.81 (5.96-7.75) | 196 | 2951 | 6.64 (5.74-7.64) | 6.89 (5.96-7.93) | 207 | 1931 | 10.72 (9.31-12.28) | 10.72 (9.31-12.28) |
| 50-54 | 204 | 2549 | 8 (6.94-9.18) | 8.03 (6.97-9.22) | 347 | 3512 | 9.88 (8.87-10.98) | 9.84 (8.83-10.94) | 504 | 3422 | 14.73 (13.47-16.07) | 14.73 (13.47-16.07) |
| 55-59 | 322 | 3512 | 9.17 (8.19-10.23) | 9.27 (8.29-10.35) | 320 | 2770 | 11.55 (10.32-12.89) | 11.75 (10.5-13.11) | 547 | 2966 | 18.44 (16.93-20.05) | 18.44 (16.93-20.05) |
| 60-64 | 328 | 2875 | 11.41 (10.21-12.71) | 11.44 (10.24-12.75) | 439 | 3468 | 12.66 (11.5-13.9) | 12.43 (11.3-13.65) | 681 | 3287 | 20.72 (19.19-22.33) | 20.72 (19.19-22.33) |
| 65-69 | 218 | 1828 | 11.93 (10.39-13.62) | 12.21 (10.64-13.95) | 376 | 2544 | 14.78 (13.32-16.35) | 15.12 (13.62-16.73) | 710 | 3063 | 23.18 (21.51-24.95) | 23.18 (21.51-24.95) |
| 70-74 | 157 | 1363 | 11.52 (9.79-13.47) | 11.69 (9.93-13.68) | 273 | 1689 | 16.16 (14.3-18.2) | 16.12 (14.26-18.15) | 484 | 1924 | 25.16 (22.96-27.5) | 25.16 (22.96-27.5) |
| 75-79 | 79 | 867 | 9.11 (7.21-11.36) | 9.11 (7.21-11.36) | 129 | 1009 | 12.78 (10.67-15.19) | 13.06 (10.9-15.53) | 328 | 1284 | 25.55 (22.86-28.46) | 25.55 (22.86-28.46) |
| ≥ 80 | 58 | 646 | 8.98 (6.82-11.61) | 8.83 (6.7-11.44) | 84 | 657 | 12.79 (10.2-15.83) | 12.73 (10.15-15.76) | 246 | 1138 | 21.62 (19-24.49) | 21.62 (19-24.49) |
| Sex | | | | | | | | | | | | |
| Male | 553 | 8327 | 6.64 (6.1-7.22) | 6.96 (6.37-7.61) | 666 | 9000 | 7.4 (6.85-7.98) | 7.75 (7.16-8.38) | 1235 | 9056 | 13.64 (12.89-14.42) | 13.64 (12.89-14.42) |
| Female | 1043 | 8767 | 11.9 (11.19-12.64) | 12.48 (11.7-13.31) | 1498 | 9600 | 15.6 (14.82-16.41) | 16.18 (15.35-17.04) | 2472 | 9959 | 24.82 (23.85-25.82) | 24.82 (23.85-25.82) |
| Level of Education | | | | | | | | | | | | |
| No formal education | 605 | 4668 | 12.96 (11.95-14.04) | 13.74 (12.57-15.02) | 740 | 4183 | 17.69 (16.44-19.01) | 18.07 (16.74-19.5) | 1193 | 4299 | 27.75 (26.2-29.37) | 27.75 (26.2-29.37) |
| Elementary school or lower | 696 | 6696 | 10.39 (9.64-11.2) | 10.83 (9.96-11.78) | 899 | 6673 | 13.47 (12.61-14.38) | 13.73 (12.82-14.7) | 1654 | 8118 | 20.37 (19.4-21.38) | 20.37 (19.4-21.38) |
| Junior high school or high school | 277 | 4877 | 5.68 (5.03-6.39) | 5.87 (5.12-6.84) | 340 | 4714 | 7.21 (6.47-8.02) | 7.17 (6.39-8.07) | 784 | 5743 | 13.65 (12.71-14.64) | 13.65 (12.71-14.64) |
| Vocational school or higher | 17 | 832 | 2.04 (1.19-3.27) | 2.45 (1.36-6.12) | 20 | 749 | 2.67 (1.63-4.12) | 2.71 (1.62-9.58) | 76 | 855 | 8.89 (7-11.13) | 8.89 (7-11.13) |
| Residence Type | | | | | | | | | | | | |
| Rural | 1357 | 12676 | 10.71 (10.14-11.29) | 11.42 (10.78-12.08) | 1672 | 12906 | 12.96 (12.34-13.59) | 13.57 (12.92-14.26) | 2864 | 13534 | 21.16 (20.39-21.95) | 21.16 (20.39-21.95) |
| Urban-Rural Fringe | 20 | 243 | 8.23 (5.03-12.71) | 7.98 (4.71-13.25) | 171 | 1707 | 10.02 (8.57-11.64) | 10.68 (9.1-12.53) | 262 | 1543 | 16.98 (14.99-19.17) | 16.98 (14.99-19.17) |
| Urban | 218 | 4139 | 5.27 (4.59-6.01) | 5.38 (4.65-6.2) | 305 | 3834 | 7.96 (7.09-8.9) | 8.19 (7.27-9.21) | 567 | 3843 | 14.75 (13.56-16.02) | 14.75 (13.56-16.02) |
| Hypertension | | | | | | | | | | | | |
| No | 1068 | 12796 | 8.35 (7.85-8.86) | 8.76 (8.22-9.33) | 1258 | 12645 | 9.95 (9.41-10.51) | 10.42 (9.85-11.03) | 1923 | 11651 | 16.51 (15.78-17.26) | 16.51 (15.78-17.26) |
| Yes | 513 | 4208 | 12.19 (11.16-13.29) | 12.37 (11.28-13.56) | 867 | 5718 | 15.16 (14.17-16.21) | 15.33 (14.3-16.42) | 1736 | 7127 | 24.36 (23.23-25.53) | 24.36 (23.23-25.53) |
| Diabetes | | | | | | | | | | | | |
| No | 1458 | 15957 | 9.14 (8.67-9.62) | 9.62 (9.11-10.15) | 1877 | 16887 | 11.12 (10.62-11.63) | 11.63 (11.1-12.18) | 3096 | 16549 | 18.71 (18.05-19.38) | 18.71 (18.05-19.38) |
| Yes | 122 | 985 | 12.39 (10.29-14.79) | 12.08 (9.92-14.67) | 262 | 1619 | 16.18 (14.28-18.27) | 16 (14.08-18.15) | 566 | 2323 | 24.37 (22.4-26.46) | 24.37 (22.4-26.46) |
| ADL Disability | | | | | | | | | | | | |
| No | 866 | 14056 | 6.16 (5.76-6.59) | 6.33 (5.89-6.8) | 1098 | 14573 | 7.53 (7.1-7.99) | 7.71 (7.25-8.19) | 2227 | 15250 | 14.6 (14-15.22) | 14.6 (14-15.22) |
| Yes | 723 | 2824 | 25.6 (23.77-27.54) | 25.19 (23.28-27.24) | 1056 | 3483 | 30.32 (28.52-32.2) | 29.55 (27.71-31.51) | 1478 | 3518 | 42.01 (39.9-44.21) | 42.01 (39.9-44.21) |
| Physical Inactivity | | | | | | | | | | | | |
| No | 564 | 5385 | 10.47 (9.63-11.37) | 10.86 (9.93-11.88) | 835 | 7490 | 11.15 (10.4-11.93) | 11.64 (10.85-12.49) | 3039 | 15954 | 19.05 (18.38-19.74) | 19.05 (18.38-19.74) |
| Yes | 108 | 1240 | 8.71 (7.14-10.52) | 8.98 (7.27-11.12) | 191 | 1542 | 12.39 (10.69-14.27) | 13.02 (11.17-15.17) | 668 | 3056 | 21.86 (20.23-23.58) | 21.86 (20.23-23.58) |

*The standardization was performed using the 2018 CHARLS population structure as the reference, adjusting for age and gender. CI = Confidence Interval; ADL = Activities of Daily Living.

Supplementary Table 2. Temporal trends of standardized knee pain prevalence across demographic/health subgroups

| Characteristics | 2011 (*n* = 17094) | | | | 2015 (*n* = 18600) | | | | 2018 (*n* = 19015) | | | |
|---|---|---|---|---|---|---|---|---|---|---|---|---|
| | **Case (*n*)** | **Pop (*n*)** | **Crude prevalence (%) [95% CI]** | **Standardized prevalence (%) [95% CI]** | **Case (*n*)** | **Pop (*n*)** | **Crude prevalence (%) [95% CI]** | **Standardized prevalence (%) [95% CI]** | **Case (*n*)** | **Pop (*n*)** | **Crude prevalence (%) [95% CI]** | **Standardized prevalence (%) [95% CI]** |
| Age group | | | | | | | | | | | | |
| 45-49 | 331 | 3454 | 9.58 (8.58-10.67) | 9.78 (8.75-10.89) | 337 | 2951 | 11.42 (10.23-12.71) | 11.8 (10.57-13.13) | 392 | 1931 | 20.3 (18.34-22.41) | 20.3 (18.34-22.41) |
| 50-54 | 291 | 2549 | 11.42 (10.14-12.81) | 11.46 (10.18-12.86) | 468 | 3512 | 13.33 (12.15-14.59) | 13.27 (12.09-14.53) | 836 | 3422 | 24.43 (22.8-26.14) | 24.43 (22.8-26.14) |
| 55-59 | 446 | 3512 | 12.7 (11.55-13.93) | 12.86 (11.69-14.11) | 426 | 2770 | 15.38 (13.95-16.91) | 15.63 (14.18-17.19) | 819 | 2966 | 27.61 (25.75-29.57) | 27.61 (25.75-29.57) |
| 60-64 | 441 | 2875 | 15.34 (13.94-16.84) | 15.38 (13.97-16.88) | 557 | 3468 | 16.06 (14.75-17.45) | 15.79 (14.51-17.16) | 1005 | 3287 | 30.57 (28.71-32.53) | 30.57 (28.71-32.53) |
| 65-69 | 291 | 1828 | 15.92 (14.14-17.86) | 16.33 (14.5-18.32) | 499 | 2544 | 19.61 (17.93-21.41) | 20.07 (18.34-21.91) | 1003 | 3063 | 32.75 (30.75-34.84) | 32.75 (30.75-34.84) |
| 70-74 | 215 | 1363 | 15.77 (13.74-18.03) | 15.98 (13.91-18.27) | 338 | 1689 | 20.01 (17.94-22.26) | 19.96 (17.88-22.2) | 653 | 1924 | 33.94 (31.39-36.65) | 33.94 (31.39-36.65) |
| 75-79 | 109 | 867 | 12.57 (10.32-15.17) | 12.57 (10.32-15.16) | 178 | 1009 | 17.64 (15.14-20.43) | 18.01 (15.46-20.87) | 459 | 1284 | 35.75 (32.55-39.17) | 35.75 (32.55-39.17) |
| ≥ 80 | 86 | 646 | 13.31 (10.65-16.44) | 13.14 (10.5-16.25) | 111 | 657 | 16.89 (13.9-20.35) | 16.82 (13.84-20.26) | 364 | 1138 | 31.99 (28.78-35.45) | 31.99 (28.78-35.45) |
| Sex | | | | | | | | | | | | |
| Male | 786 | 8327 | 9.44 (8.79-10.12) | 9.87 (9.16-10.63) | 909 | 9000 | 10.1 (9.45-10.78) | 10.43 (9.75-11.15) | 1938 | 9056 | 21.4 (20.46-22.37) | 21.4 (20.46-22.37) |
| Female | 1424 | 8767 | 16.24 (15.41-17.11) | 17 (16.08-17.96) | 2005 | 9600 | 20.89 (19.98-21.82) | 21.5 (20.55-22.49) | 3593 | 9959 | 36.08 (34.91-37.28) | 36.08 (34.91-37.28) |
| Level of Education | | | | | | | | | | | | |
| No formal education | 850 | 4668 | 18.21 (17.01-19.48) | 19.06 (17.68-20.54) | 965 | 4183 | 23.07 (21.64-24.57) | 23.63 (22.1-25.26) | 1633 | 4299 | 37.99 (36.17-39.87) | 37.99 (36.17-39.87) |
| Elementary school or lower | 948 | 6696 | 14.16 (13.27-15.09) | 14.81 (13.79-15.91) | 1167 | 6673 | 17.49 (16.5-18.52) | 17.74 (16.71-18.84) | 2433 | 8118 | 29.97 (28.79-31.19) | 29.97 (28.79-31.19) |
| Junior high school or high school | 389 | 4877 | 7.98 (7.2-8.81) | 8.22 (7.3-9.35) | 481 | 4714 | 10.2 (9.31-11.16) | 10.3 (9.35-11.37) | 1306 | 5743 | 22.74 (21.52-24.01) | 22.74 (21.52-24.01) |
| Vocational school or higher | 22 | 832 | 2.64 (1.66-4) | 2.92 (1.75-6.59) | 35 | 749 | 4.67 (3.25-6.5) | 4.65 (3.2-11.47) | 159 | 855 | 18.6 (15.82-21.72) | 18.6 (15.82-21.72) |
| Residence Type | | | | | | | | | | | | |
| Rural | 1863 | 12676 | 14.7 (14.04-15.38) | 15.64 (14.9-16.41) | 2224 | 12906 | 17.23 (16.52-17.96) | 17.89 (17.14-18.67) | 4142 | 13534 | 30.6 (29.68-31.55) | 30.6 (29.68-31.55) |
| Urban-Rural Fringe | 28 | 243 | 11.52 (7.66-16.65) | 10.86 (7.03-16.6) | 230 | 1707 | 13.47 (11.79-15.33) | 14.12 (12.3-16.19) | 418 | 1543 | 27.09 (24.56-29.82) | 27.09 (24.56-29.82) |
| Urban | 318 | 4139 | 7.68 (6.86-8.58) | 7.83 (6.95-8.81) | 436 | 3834 | 11.37 (10.33-12.49) | 11.67 (10.57-12.88) | 944 | 3843 | 24.56 (23.02-26.18) | 24.56 (23.02-26.18) |
| Hypertension | | | | | | | | | | | | |
| No | 1519 | 12796 | 11.87 (11.28-12.48) | 12.47 (11.83-13.15) | 1728 | 12645 | 13.67 (13.03-14.33) | 14.17 (13.5-14.87) | 2977 | 11651 | 25.55 (24.64-26.49) | 25.55 (24.64-26.49) |
| Yes | 671 | 4208 | 15.95 (14.76-17.2) | 16.04 (14.79-17.38) | 1139 | 5718 | 19.92 (18.78-21.11) | 20.06 (18.88-21.3) | 2486 | 7127 | 34.88 (33.52-36.28) | 34.88 (33.52-36.28) |
| Diabetes | | | | | | | | | | | | |
| No | 2019 | 15957 | 12.65 (12.11-13.22) | 13.29 (12.69-13.92) | 2524 | 16887 | 14.95 (14.37-15.54) | 15.49 (14.87-16.12) | 4627 | 16549 | 27.96 (27.16-28.78) | 27.96 (27.16-28.78) |
| Yes | 170 | 985 | 17.26 (14.76-20.06) | 16.72 (14.16-19.7) | 356 | 1619 | 21.99 (19.76-24.4) | 21.85 (19.59-24.34) | 842 | 2323 | 36.25 (33.84-38.78) | 36.25 (33.84-38.78) |
| ADL Disability | | | | | | | | | | | | |
| No | 1289 | 14056 | 9.17 (8.68-9.69) | 9.4 (8.87-9.97) | 1558 | 14573 | 10.69 (10.17-11.24) | 10.81 (10.27-11.37) | 3577 | 15250 | 23.46 (22.69-24.24) | 23.46 (22.69-24.24) |
| Yes | 905 | 2824 | 32.05 (29.99-34.2) | 31.74 (29.58-34.03) | 1338 | 3483 | 38.42 (36.38-40.53) | 37.52 (35.43-39.73) | 1952 | 3518 | 55.49 (53.05-58) | 55.49 (53.05-58) |
| Physical Inactivity | | | | | | | | | | | | |
| No | 748 | 5385 | 13.89 (12.91-14.92) | 14.32 (13.25-15.47) | 1113 | 7490 | 14.86 (14-15.76) | 15.42 (14.51-16.39) | 4565 | 15954 | 28.61 (27.79-29.46) | 28.61 (27.79-29.46) |
| Yes | 141 | 1240 | 11.37 (9.57-13.41) | 12.38 (10.29-14.9) | 263 | 1542 | 17.06 (15.06-19.25) | 17.88 (15.69-20.36) | 966 | 3056 | 31.61 (29.65-33.67) | 31.61 (29.65-33.67) |

*The standardization was performed using the 2018 CHARLS population structure as the reference, adjusting for age and gender. CI = Confidence Interval; ADL = Activities of Daily Living.
